# Subclonal β-catenin/YAP signaling heterogeneity accelerates ovarian cancer metastasis through a senescence-associated secretory phenotype

**DOI:** 10.1038/s41419-026-08737-7

**Published:** 2026-04-23

**Authors:** Sally K. Y. To, Yibin Yang, Zeyu Shi, Katie S. W. Fung, Sook Ling Lai, Joshua J. X. Li, Philip P. C. Ip, Alice S. T. Wong

**Affiliations:** 1 School of Biological Sciences, University of Hong Kong, Hong Kong, China; 2 Laboratory for Synthetic Chemistry and Chemical Biology Limited, Hong Kong Science and Technology Parks, Hong Kong, China; 3 Department of Pathology, Queen Mary Hospital, University of Hong Kong, Hong Kong, China; 4 Clincial Oncology Medical Center, HKU-Shenzhen Hospital, Shenzhen, China

**Keywords:** Ovarian cancer, Metastasis, Cell signalling, Post-translational modifications

## Abstract

Intratumoral heterogeneity in metastatic cancers complicates effective treatment, as distinct tumor subclones display varying drug sensitivities and metastatic capabilities, interacting through mechanisms that remain poorly understood. In this study, we utilized an isogenic ovarian cancer cell pair distinguished by differential β-catenin signaling: non-metastatic (NM) cells with low β-catenin and highly metastatic (HM) cells with elevated β-catenin signaling. Co-engrafting NM and HM cells synergistically enhances peritoneal metastasis compared to either subclone alone. Immunohistochemical analysis revealed a mosaic β-catenin expression in mixed tumors, recapitulating the heterogeneity observed in clinical ovarian cancer cases. Notably, both NM cells and β-catenin-knockout HM cells exhibited an intrinsic senescence-associated secretory phenotype (SASP), mechanistically driven by Hippo/YAP signaling, which was suppressed by β-catenin in HM cells. This phenotype in turn facilitated the metastatic dissemination of β-catenin-high subclones through paracrine signaling. Importantly, mixed in vivo tumors were more susceptible to treatment with fisetin, a senolytic agent, and YAP inhibition, leading to marked reduction in metastatic burden. Overall, our findings demonstrate the pre-existence of senescence-like tumor cells in untreated conditions and reveal a novel cooperative mechanism in which β-catenin heterogeneity facilitates ovarian cancer progression through SASP-mediated subclonal interactions. These results provide a basis for targeted therapeutic strategies to disrupt intratumoral communication and improve treatment outcomes in metastatic ovarian cancer.

## Main

Intratumoral heterogeneity, characterized by the co-existence of phenotypically and functionally distinct subpopulations within a single neoplastic lesion, represents a fundamental challenge in cancer treatment. Numerous studies have demonstrated that this diversity can drive rapid tumor progression, treatment resistance, and metastasis, presenting a significant challenge for the development of effective cancer therapies and limiting reproducible prognostic classifications [1, 2]. Despite widespread recognition of its clinical importance, our understanding of how heterogeneous subclones interact to promote malignancy remains limited. The majority of cancer research has focused on studying the dominant tumor subclone which are capable of evading clinical interventions and seeding new metastatic foci, often neglecting the contributions and implications of minor subpopulations. While genomic studies have mapped clonal architecture, they often overlook the dynamic functional relationships and interdependency between subpopulations that collectively drive tumor progression [3, 4]. These relationships are crucial for identifying vulnerabilities that can be exploited for therapeutic benefit. Recent evidence suggests that tumor subclones do not merely compete but can actively cooperate, playing a direct functional role rather than just providing a reservoir of variants that allows rapid response [5–7]. It is also important to note that subclonal interaction could result in group behaviors and the emergence of new capabilities that cannot be predicted by studying the properties of individual subpopulations [8].

Advanced ovarian cancer is the most lethal of all gynecological malignancies, characterized by widespread peritoneal metastasis and the accumulation of malignant ascites. Despite extensive efforts, current therapies remain largely ineffective, resulting in a dismal 5-year survival rate of <25% [9]. Underpinning this poor prognosis is the extensive intratumoral heterogeneity, both spatially within the primary tumor and temporally as the ovarian cancer progresses [10]. This heterogeneity is manifested across a diverse array of molecular and phenotypic features, such as transcriptomic profiles, copy number variations, cellular phenotypes, proliferative indices and homologous recombination deficiency scores [11, 12]. However, these observations have not yet been effectively translated into actionable therapeutic strategies.

Our previous work established β-catenin overactivation as a key driver of metastasis and therapy resistance in ovarian cancer [13–15]. Indeed, heterogeneous expression of β-catenin has been detected in various types of tumors [16]. β-catenin-high and -low subpopulations also coexist in primary ovarian cancers and ascites [17–19]. Building on these findings, we now demonstrate that spatially heterogeneous β-catenin expression creates functionally interdependent subclones that cooperatively drive metastasis. We identified a novel mechanism whereby β-catenin-low subclones, through activation of Hippo/YAP signaling, develop a senescence-associated secretory phenotype (SASP) that paradoxically enhances the metastatic potential of neighboring β-catenin-high cells.

## Results

### NM cells accelerate metastasis of metastatic cells via soluble factors

**Fig. 1:**
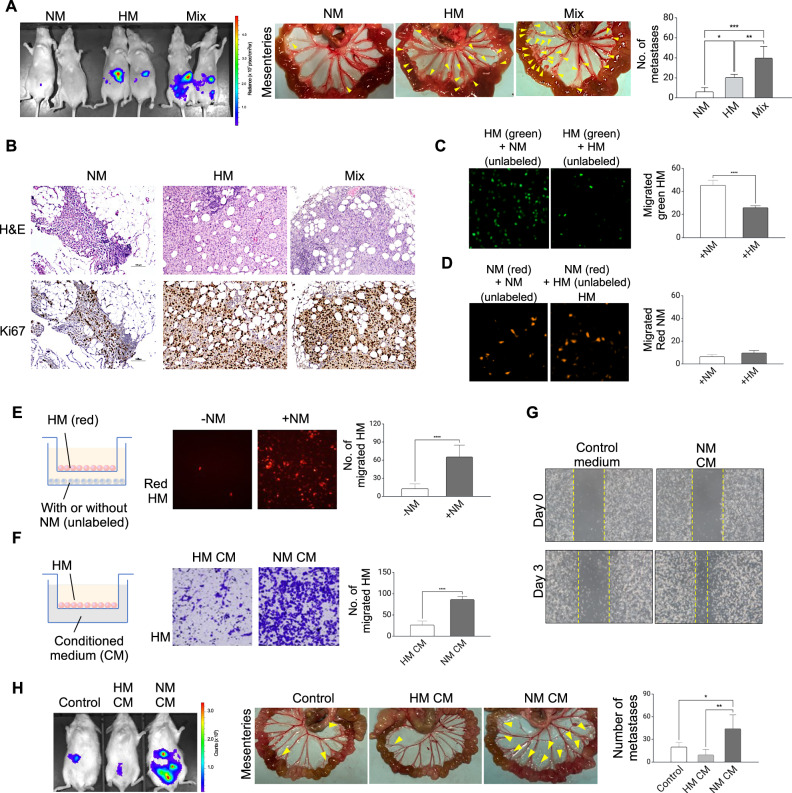
NM potentiates the metastatic phenotype of HM through secreted factors. **A** HM and NM cells were intraperitoneally injected into mice (*n* = 4). Peritoneal metastasis was monitored by bioluminescence imaging of luciferase activity (left). Metastatic nodules (yellow arrowheads) were photographed (middle) and counted (right). **B** Metastatic tumors were stained with hematoxylin and eosin (H&E) for histological analysis and immunostained with the proliferative marker Ki67. **C** HM cells (labeled with cell tracker green) were placed in the upper chamber and allowed to migrate in the presence of unlabeled NM or HM. Migrated green HM cells were captured by fluorescence microscope and counted. **D** NM cells (labeled with cell tracker red) were placed in the upper chamber and allowed to migrate in the presence of unlabeled NM or HM in direct contact. Migrated red NM cells were captured by fluorescence microscope and counted in five random fields of view. **E** HM (red) were allowed to migrated in the absence or presence of NM cells seeded at the lower chamber. Migrated HM cells were captured by fluorescence microscope and counted. **F** After a scratch wound was formed, HM cells were cultured in control serum-free medium or NM conditioned medium (CM) and a wound healing assay was performed. **G** Migrated cells were stained with crystal violet, and captured HM cells were allowed to migrate in the absence or presence of NM CM. Migrated cells were stained with crystal violet and captured. **H** HM cells were i.p. injected into immunodeficient mice (*n* = 4). One week after tumor inoculation, mice were treated with concentrated regular medium, concentrated HM CM or concentrated NM CM (200 μl every 2 days) for two weeks. Peritoneal metastasis was monitored by bioluminescence imaging of luciferase activity (left); and the number of metastasis nodules was photographed and counted (right). Data are presented as mean ± SD. *P < 0.05, **P < 0.01, ***P < 0.001.

To investigate the functional significance of tumor heterogeneity in metastasis, we employed a well-controlled and clinically relevant model comprising non-metastatic (NM) and highly metastatic (HM) sublines derived from the HEYA8 ovarian cancer cell line that we had previously established [14]. This cell line pair, characterized by low β-catenin signaling in NM and high β-catenin signaling in HM, has provided a valuable platform for identifying key molecules involved in tumor metastasis [14, 15]. In this study, intraperitoneal injection was chosen over orthotopic injection to directly model peritoneal seeding, the dominant pattern in advanced ovarian cancer, while allowing tight control over tumor cell number, timing, and subpopulation composition. Orthotopic injection, although capable of generating a primary ovarian mass with spontaneous metastasis, introduces stromal and anatomical constraints, adding variability that may obscure early direct interactions between defined subpopulations. To create a controlled co-existence of metastasis-high and metastasis-low subpopulations in the same microenvironment so that their behaviors and competitive dynamics could be compared under identical in vivo conditions, a 1:1 mix was selected as an experimentally tractable, reproducible condition that guarantees the presence of both subpopulations at comparable starting frequencies. Indeed, this ratio is commonly employed in assessing subclonal interactions [7, 20, 21]. NM cells, HM cells, or a 1:1 mixture of both were first injected into mice intraperitoneally. Metastatic burdens were assessed by in vivo bioluminescence imaging of luciferase-labeled cells and quantification of the number of metastatic foci formed in the mesentery. NM cells alone showed no detectable metastasis, while the admixture of HM and NM cells significantly promoted peritoneal metastasis compared to injection of HM cells alone (Fig. 1A). These findings were further confirmed by H&E staining and increased expression of the proliferative marker Ki67, as determined by immunohistochemistry (Fig. 1B). These results suggest that interaction between NM and HM could promote metastatic colonization. To determine whether NM cells promote the metastasis of HM cells or vice versa, we co-injected luciferase-labeled NM and unlabeled HM cells into mice. Interestingly, only HM cells were present at the time of harvest, suggesting that NM cells may play a supportive role during the early stages of metastatic progression (Fig. S1A). We also performed the reciprocal labeling experiment, in which HM cells were stably transduced with luciferase while NM cells were unlabeled. In this setting, luciferase-expressing HM cells displayed faster in vivo growth and peritoneal colonization when co-injected with NM cells (Fig. S1B), consistent with our original hypothesis regarding the higher metastatic fitness of the HM subpopulation in the presence of NM cells. Collectively, these data demonstrate that the NM subclone can strongly enhance the metastatic behavior of the HM subclone in ovarian cancer, underscoring the functional significance of tumor heterogeneity in driving metastatic progression.

### A SASP of NM mediates the metastatic cooperation

We next conducted migration assays to explore the interactions between the two subclones in vitro. To distinguish the subclones in heterotypic mixtures, we used cell tracker dye to fluorescently label NM and HM cells. We observed that the presence of NM cells, but not HM cells, significantly enhanced the migration of HM cells labeled with green fluorescence (Fig. 1C). In contrast, the presence of neither HM nor NM cells had any effect on the migration of NM cells labeled with red fluorescence (Fig. 1D). These are in support of the in vivo data that NM promotes HM metastasis but not vice versa. To investigate the requirement for direct cell-cell contact in HM-NM crosstalk, a two-chamber culture system was employed. This system allowed for shared culture medium but prevented physical interaction between HM and NM cells. Results demonstrated that NM induced HM migration in the absence of direct contact (Fig. 1E). To investigate this contact-independent mechanism, conditioned medium (CM) was collected from NM cells. Addition of NM-derived CM to HM cells resulted in a significant increase in migratory phenotype compared to HM-derived CM or control medium, suggesting the involvement of soluble factors secreted by NM cells (Fig. 1F, G). To validate the in vivo relevance of these findings, HM-bearing mice were treated with NM-derived CM. As shown in Fig. 1H, secreted factors from NM cells significantly promoted HM metastasis compared to control medium and HM CM, confirming the ability of soluble factors derived from NM cells to enhance HM metastasis.

**Fig. 2:**
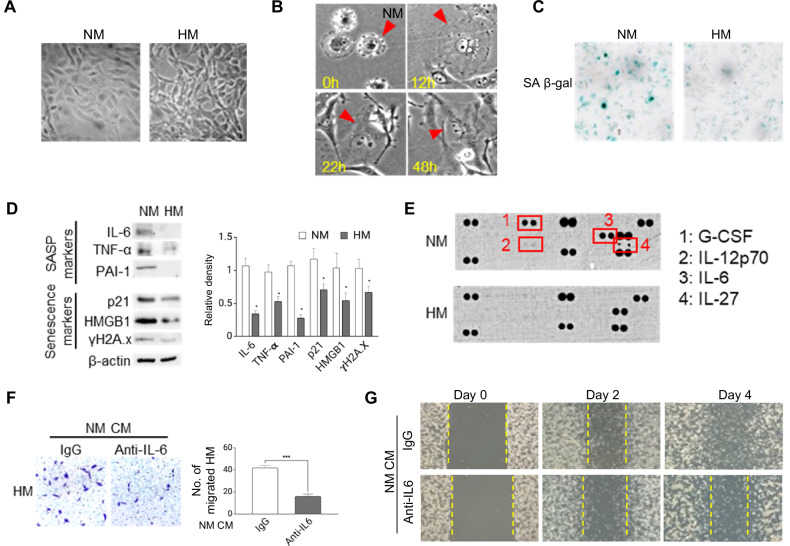
NM exhibits a senescence-associated secretory phenotype (SASP). **A** Representative images of morphology features of NM and HM. **B** NM cells were tracked by single-cell time-lapse microscopy over 48 h. Representative images of a non-mitotic NM cell (arrow) were shown. **C** NM and HM cells were stained for senescence-associated β-galactosidase (SA-β-gal) (blue). **D** Western blots of senescence markers (p21, HMGB1, and γH2A.x) and senescence-associated secretory phenotype (SASP) factors (IL-6, TNF-α, and PAI-1 in NM compared to HM, with β-actin as the loading control. **E** Conditioned medium (CM) from HM and NM was analysed with cytokine array. **F**, **G** IL-6 neutralization inhibits NM CM-induced HM migration in (**F**) migration assays and (**G**) wound-healing assays. Data are presented as mean ± SD. *P < 0.05, **P < 0.01, ***P < 0.001.

NM cells exhibited a distinct morphology, characterized by a larger and more flattened shape compared to HM cells under standard culture conditions (Fig. 2A). Time-lapse microscopy indicated that most NM cells were non-mitotic over a 48-hour period (Fig. 2B), suggesting a potential senescent-like state. To test this, we performed staining for senescence-associated β-galactosidase (SA-β-gal), a commonly used marker of cellular senescence. While HM cells showed minimal SA-β-gal staining, NM cells exhibited significantly increased staining (Fig. 2C), supporting the notion of senescence in NM cells. Further analysis using Western blotting confirmed elevated levels of canonical senescence markers (p21, HMGB1, and γH2A.x) and SASP factors (IL-6, TNF-α, and PAI-1) in NM compared to HM (Fig. 2D). Cytokine array analysis revealed an upregulation of SASP-associated cytokines, including IL-6, G-CSF, IL-12p70, and IL-27, in NM cells (Fig. 2E). These cytokines are known to be abundant in ovarian cancer ascites [22–25]. We consistently observed an upregulation of various genes encoding SASPs, including chemokines, cytokines, growth factors, extracellular matrix proteins, and other factors in NM compared to HM, based on RNA-seq data (Supplementary Table 1). Given the established role of IL-6 as a key SASP cytokine [26], we investigated its impact on HM migration. Neutralization of IL-6 partially inhibited HM migration induced by NM-derived CM (Fig. 2F, G), suggesting a role for SASP in promoting HM aggressiveness.

### β-catenin suppresses senescence-like phenotypes

**Fig. 3:**
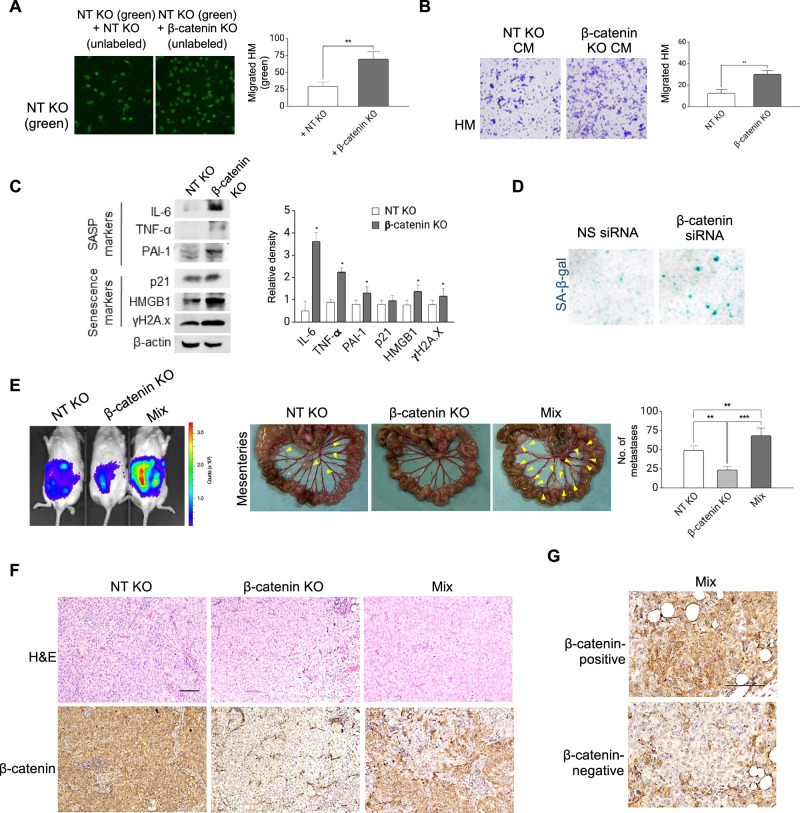
β-catenin heterogeneity promotes metastasis. **A** HM cells with a non-targeting knockout (NT KO) (labeled with cell tracker green) were placed in the upper chamber and allowed to migrate in the presence of unlabeled NT KO or β-catenin KO HM cells in direct contact. Migrated green NT KO cells were captured by fluorescence microscope and counted in five random fields of view. **B** NT KO cells were allowed to migrate in the absence or presence of conditioned medium (CM) from β-catenin KO HM cells. Migrated cells were stained with crystal violet and captured. **C** Western blots of senescence and SASP markers in β-catenin KO compared to NT KO cells. b-actin serves as the loading control. **D** Cells were transfected with non-specific (NS) or β-catenin siRNA and stained for SA-b-gal (blue). **E** NT KO or β-catenin KO or 1:1 mixture of cells were intraperitoneally injected into mice (*n* = 4). Peritoneal metastasis was monitored by bioluminescence imaging of luciferase activity (left); the number of metastatic nodules was photographed (middle) and counted (right). **F** Metastatic tumors were stained with H&E for histological analysis and immunostained with β-catenin. Scale bar = 100 μM. **G** High magnification of β-catenin-positive and -negative regions in the mix tumors. Scale bar = 100 μM. Data are presented as mean ± SD. *P < 0.05, **P < 0.01, ***P < 0.001.

We next investigated whether the heightened β-catenin signaling might suppress the SASP in HM cells. β-catenin knockout (KO) and non-targeting (NT) KO control cells were generated in HM using CRISPR/Cas9 genome editing. Successful knockout of β-catenin was confirmed by Western blot analysis (Fig. S2A). NT KO cells exhibited significantly increased migration when co-cultured with β-catenin KO cells compared to when cultured with NT KO cells under non-contact conditions (Fig. 3A). Furthermore, HM cells exposed to CM from β-catenin KO cells displayed enhanced migration compared to those exposed to CM from NT KO cells (Fig. 3B), suggesting a role for soluble factors. Consistent with these findings, β-catenin KO cells showed elevated expression of SASP and senescence markers, as well as SA-β-gal staining, as compared to NT KO cells (Fig. 3C, D).

To investigate the in vivo relevance of heterogenous β-catenin expression in metastasis, β-catenin KO and NT KO HM cells were injected alone or in 1:1 mixture intraperitoneally into mice. β-catenin KO cells exhibited reduced metastatic potential compared to NT KO cells (Fig. 3E), consistent with our previous studies. Notably, co-injection of β-catenin KO and NT KO cells resulted in significantly increased metastatic burden compared to injection of either cell clone alone (Fig. 3E), indicating a potential for clonal interactions between β-catenin-positive and -negative cells. The presence of β-catenin-positive and -negative cells in the mixed tumor was demonstrated by immunohistochemistry (Fig. 3F, G). Moreover, HM cells expressing non-specific or β-catenin shRNA were able to form heterotypic spheroids (Fig. S2B). These findings together suggest that β-catenin could negatively regulate SASP, and that SASP plays a critical role in promoting tumor metastasis of the heterogenous tumor.

**Fig. 4:**
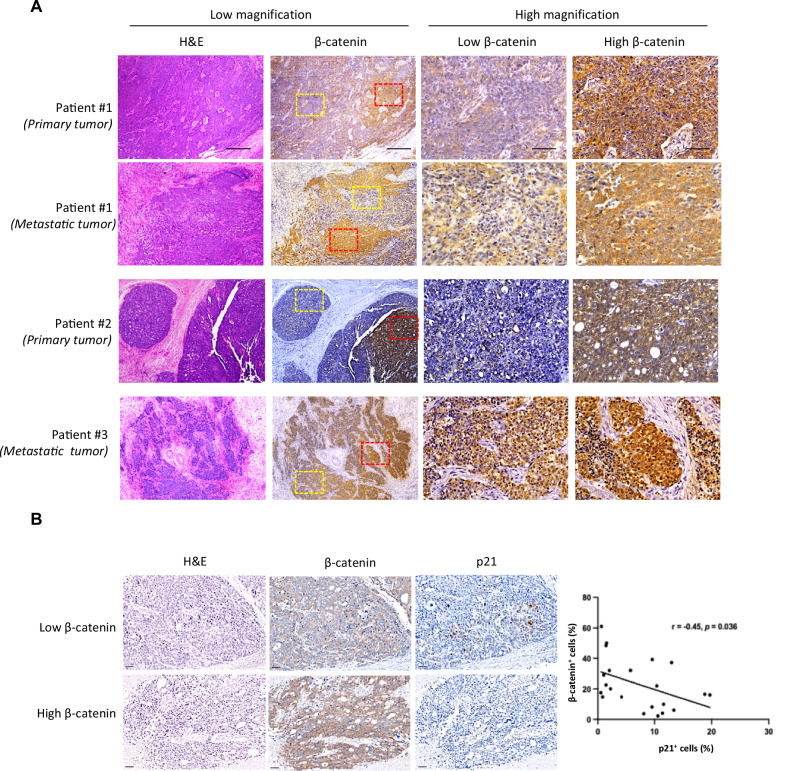
Clinical ovarian cancer tumors showed intratumoral heterogeneity in β-catenin expression. **A** H&E staining and immunohistochemistry of β-catenin were performed on tumor sections from three different cases of human high-grade serous ovarian cancer. Tumor 1 & 3 were from the primary site, and tumor 2 was from the omental metastatic site. Area with low (yellow box) and high (red box) expression of β-catenin were shown in high magnification. Scale bar for low magnification field: 500 μm; for high magnification field: 10 μm. **B** Consecutive tumor sections from HGSOC patients (*n* = 11) were stained for β-catenin and p21, and the number of positively stained tumor cells per mm² was quantified in matched fields using QuPath. Representative fields illustrating β-catenin-high and β-catenin-low regions from the same patient are shown. The percentage of β-catenin-positive tumor cells was then correlated with the percentage of p21-positive tumor cells using Pearson’s correlation analysis. Scale bar = 100 μm.

To assess clinical relevance, we performed immunohistochemistry on tumor samples from patients with high-grade serous ovarian cancer, the most prevalent subtype. Within the same microscopic sections from both primary ovarian tumors and matched omental metastases, we observed heterogeneous β-catenin expression (Figs. 4A and S3A). Since β‑catenin signaling is well known to drive a proliferative oncogenic program, and prior work from our group and others indicates that low β‑catenin-expressing tumors generally grow more slowly than their high β‑catenin-expressing counterparts in experimental models, one might anticipate an enrichment of β‑catenin‑high cells in patient tumors. However, tumor growth in patients is indeed also shaped by inter‑patient differences and microenvironmental influences that extend beyond β‑catenin levels alone. To better capture this complexity, we quantified the relative proportions of β‑catenin‑low and β‑catenin‑high cells across patient tumors, revealing β‑catenin‑low: β‑catenin‑high ratios of 1:0.11–0.56 in primary tumors and 1:0.46–1.26 in metastatic lesions (Fig. S3B). These data indicate that the degree of heterogeneity varies substantially between tumors and, despite deviations in individual cases (such as patient 1, where a β‑catenin‑low region appears larger in area), β‑catenin‑low and β‑catenin‑high cells consistently coexist within tumors rather than forming uniformly monomorphic lesions. Organoids derived from patient ascites were also stained and revealed distinct populations of low and high-β-catenin-expressing cancer cells, mirroring the heterogeneity seen in vivo (Fig. S3C). In addition, immunostaining for the senescence marker p21 showed that regions with low β-catenin expression were associated with a higher abundance of senescent tumor cells in patient specimens (Fig. 4B). Taken together, these findings provide clinical evidence for intratumoral β-catenin heterogeneity and its association with senescence-like phenotypes.

### β-catenin inhibits Hippo/YAP signaling to suppress SASP

**Fig. 5:**
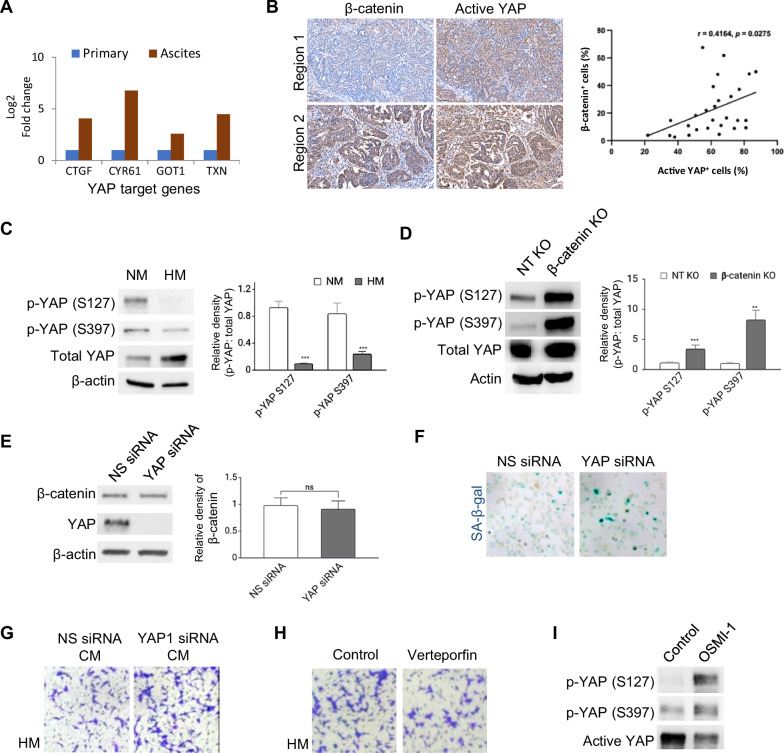
β-catenin inhibits Hippo/YAP signaling to suppress SASP. **A** Proteomic analysis comparing HM- derived primary tumor and metastatic ascitic cells in an orthotopic model showed upregulation of several YAP (CTGF, CYRB1, GOT1, and TXN) in ascitic cells. **B** Consecutive tumor sections from HGSOC patients (*N* = 11) were stained for β-catenin and active YAP. Representative images of two regions of the same tumor were shown. The number of positively stained tumor cells per mm² was quantified in matched fields using QuPath. The percentage of β-catenin-positive tumor cells was then correlated with the percentage of active YAP-positive tumor cells using Pearson’s correlation analysis. Scale bar = 100 μm. **C** Western blots of phosphorylated YAP (p-YAP) at Serine 127 (S127) or Serine 397 (S397) and total YAP in NM cells compared to HM cells. **D** Western blots of p-YAP and total YAP in β-catenin KO cells compared to NT KO cells. **E** HM cells were transfected with non-specific (NS) or YAP siRNA, followed by a western blot of YAP and β-catenin. **F** Cells were treated with senescence-associated β-galactosidase (SA-β-gal) (blue). **G** HM cells were allowed to migrate in the absence or presence of conditioned medium (CM) collected from HM transfected with NS or YAP siRNA. Migrated cells were fixed, stained with crystal violet and captured. **H** HM treated with vehicle control or verteporfin (YAP inhibitor). Migrated cells were stained with crystal violet and captured. **I** Western blots of p-YAP and active YAP (non-phosphorylated) in NT KO cells treated with or without 20 nM OSMI-1 (O-GlcNAc transferase inhibitor). Actin serves as the loading control. Data are presented as mean ± SD. *P < 0.05, **P < 0.01, ***P < 0.001.

The Hippo signaling pathway maintains tissue homeostasis and prevents uncontrolled cell expansion by inducing YAP phosphorylation, which subsequently inhibits its nuclear localization and prevents the activation of oncogenes. In our previous proteomic analysis comparing HM-derived primary tumor and metastatic ascitic cells in an orthotopic mouse model, we found an upregulation of several YAP (yes-associated protein) target genes (CTGF, CYRB1, GOT1, and TXN) in ascitic cells, suggesting potential downregulation of Hippo signaling during metastasis (Fig. 5A). Given the established crosstalk between β-catenin and Hippo signaling pathways [27], we next examined the relationship between β-catenin and YAP. Immunohistochemistry revealed a heterogeneous and positively correlated pattern of active YAP and β-catenin signals in patient tumors (Fig. 5B). Consistently, Western blot analysis demonstrated increased levels of the inactive phosphorylated forms of YAP (p-YAP; S127 and S397) in NM compared to HM cells (Fig. 5C), as well as in β-catenin KO compared to NT KO cells (Fig. 5D). These results suggest a regulatory role of β-catenin on Hippo signaling. On the other hand, YAP silencing did not affect β-catenin expression (Fig. 5E), indicating that β-catenin acts upstream of YAP in our model. We next investigated whether YAP could regulate senescent phenotypes. YAP silencing increased SA-β-gal staining HM (Fig. 5F). The addition of CM derived from YAP-silenced HM cells led to increased migration of HM cells compared to the CM derived from control cells (Fig. 5G). Verteporfin is a small molecule which inhibits YAP-TEAD-dependent transcription and has shown anti-tumor activity in multiple models. Its established clinical safety as an approved drug for macular degeneration further supports the translational potential of YAP targeting [28]. Treatment of HM with verteporfin, suppressed migration induced by NM CM (Fig. 5H). O-linked-β-N-acetylglucosamine glycosylation (O-GlcNAcylation) is a posttranslational modification that can antagonize phosphorylation by competing for the same serine and threonine sites on target proteins. O-GlcNAcylation of YAP has been shown to disrupt its interaction with the upstream kinase LATS1, preventing phosphorylation and promoting its transcriptional activity [29]. Consistently, inhibition of O-GlcNAcylation with OSMI-1 increased p-YAP in NT KO cells (Fig. 5I), suggesting that O-GlcNAcylation may influence β-catenin-associated YAP regulation, although the off-target and global effects of O-GlcNAcylation inhibition could not be excluded. Taken together, these data suggest that β-catenin/YAP signaling may negatively regulate senescence phenotypes.

### Senotherapy or YAP inhibition suppressed metastasis of the heterogeneous tumor

**Fig. 6:**
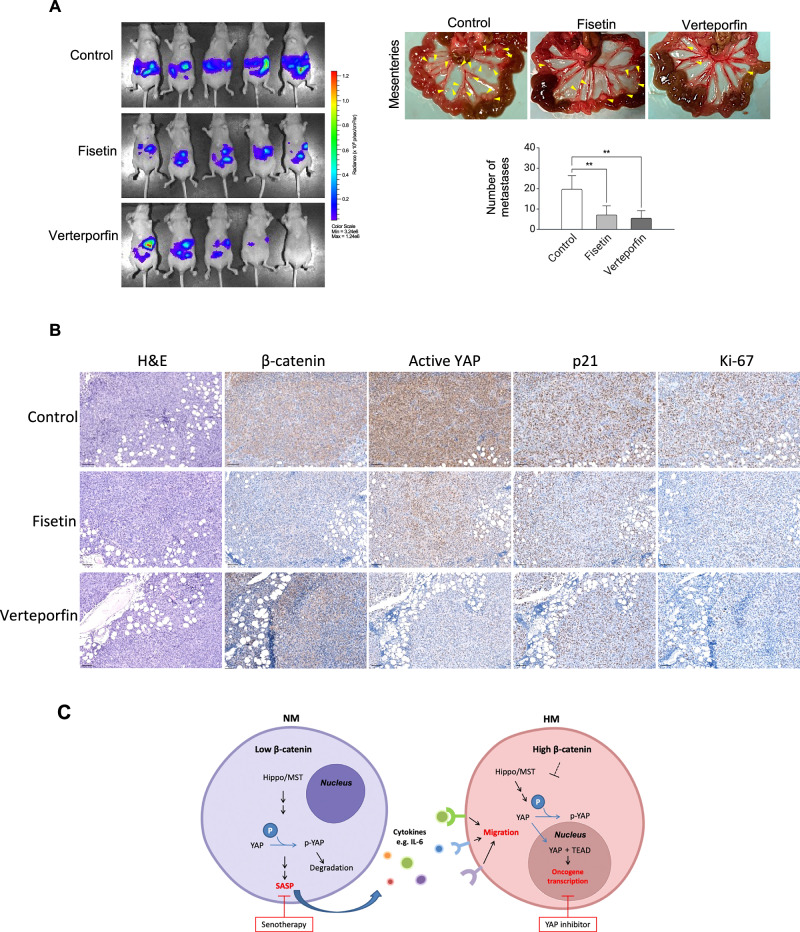
Senotherapy with fisetin or YAP inhibition suppressed metastasis. **A** NT and β-catenin KO cells in a 1:1 mixture were intraperitoneally (i.p.) injected into mice (*n* = 5). After 5 days of tumor injection, mice were i.p. treated with vehicle control, fisetin or verteporfin once every 2 days. Peritoneal metastasis was monitored by bioluminescence imaging of luciferase activity (left); the number of metastatic nodules was photographed and counted (right). **B** Metastatic tumors were stained with H&E for histological analysis and immunostained with β-catenin, active YAP, p21, and Ki67. Scale bar = 100 μM. **C** Schematic diagram of the proposal heterogeneous crosstalk between NM and HM to promote metastasis. Data are presented as mean ± SD. **P < 0.01.

To explore the therapeutic potential of targeting our proposed pathway, we employed fisetin, a naturally occurring flavonoid with potent senolytic properties [30], and verteporfin. Both verteporfin and fisetin significantly inhibited the migration of HM cells pre-incubated with β-catenin KO-derived CM (Fig. 6A). To evaluate the in vivo anti-metastatic effects, mice were intraperitoneally injected with a mixture of β-catenin KO and control HM cells. Treatment with either fisetin or verteporfin resulted in decreased metastatic formation in mice with heterogeneous tumors compared to vehicle control (Fig. 6A). Immunohistochemistry revealed more homogeneous expression of β-catenin after both treatments (Fig. 6B). These findings strongly suggest that targeting the β-catenin/YAP signaling pathway and SASP may represent a promising therapeutic strategy for metastatic ovarian cancer patients. Our proposed signaling in the subclonal crosstalk has been summarized in Fig. 6C.

## Discussion

Intratumoral heterogeneity represents a fundamental challenge in the effective treatment of cancer, complicating both diagnosis and therapeutic interventions. This complexity is particularly pronounced when considering the differential behaviors of tumor subclones during various stages of cancer progression. While the majority of current research emphasizes the impact of heterogeneity on primary tumor growth, the implications of such variability on the metastatic cascade remain largely underexplored. Our previous investigations have illuminated distinct signaling profiles in HM cells relative to their isogenic NM counterparts derived from the same parental lineage [14]. Specifically, we identified activated β-catenin signaling in HM cells, reinforcing the hypothesis of pre-existing subclonal diversity linked to disparate β-catenin activity within the bulk tumor population. This observation raises critical questions regarding the functional consequences of intratumoral β-catenin heterogeneity, particularly its role in fostering tumor aggressiveness and the precise mechanisms through which this occurs—issues that have yet to be thoroughly elucidated. Phenotypic plasticity is an important consideration when modeling metastasis in vivo using established cancer cell lines. To limit the impact of phenotypic drift on the validity of our in vivo conclusions, we routinely confirmed that HM and NM cells, generated by single-cell cloning and expanded under identical culture conditions after being isolated from the polyclonal HEYA8 parental lines, maintained distinct morphology and differential expression of key EMT markers for at least 10 passages (Fig. S4A). They also retained their differential metastatic potential in independent in vivo experiments, arguing against rapid convergence to a shared phenotypic equilibrium within the experimental time frame. Under these defined conditions, HM and NM behave as stable, functionally distinct metastasis-high and metastasis-low subpopulations rather than rapidly equilibrating mixtures, supporting the robustness of our in vivo model and its derived conclusions.

We have previously shown that HM cells exhibit a more mesenchymal phenotype, whereas NM cells retain more epithelial features [31]. Such differences in epithelial-mesenchymal state and in cell-cell and cell-matrix adhesion could, in principle, modulate how HM and NM cells interact with each other and with peritoneal surfaces, and thus represent a potential confounding variable. However, our non‑contact migration assays and in vivo experiments using CM indicate a predominant contribution of soluble, adhesion‑independent signals. Accordingly, while effects arising from differential adhesion cannot be fully excluded, the data support that a central component of the cooperative interaction between HM and NM cells is mediated by paracrine signaling.

Both the β-catenin and Hippo signaling pathways are pivotal regulators of cellular processes, including proliferation, differentiation, and maintenance of tissue homeostasis. Aberrant activation of these pathways has been recognized as a contributing factor in the onset and progression of cancer. Notably, while the oncogenic roles of β-catenin in promoting stemness and epithelial-mesenchymal transition are well-established [32], our findings reveal a paradox where β-catenin-low subclones can enhance metastasis by secreting SASP factors and this secretion non-cell autonomously facilitates the dissemination of β-catenin-high cells. These data suggest that subclonal dynamics can generate collective malignant behaviors, further complicating the landscape of intratumoral heterogeneity.

This interplay is intricately linked to the Hippo pathway, particularly through its influence on the YAP transcriptional co-activator. When Hippo signaling is inhibited, YAP can translocate to the nucleus and interact with various transcription factors to drive oncogene expression. Heterogeneous YAP signaling has been proposed to play a pivotal role in determining cell differentiation and chemoresistance, as demonstrated in models of small cell lung cancer [33]. The regulatory dynamics between β-catenin and Hippo signaling are complex [27]. For instance, Hippo signaling was shown to inhibit the nuclear translocation of β-catenin [34, 35], while β-catenin could upregulate YAP expression via direct interaction with the YAP promoter [36]. Moreover, existing literature suggests that β-catenin can form complexes with YAP to drive tumor progression and sustain characteristics of cancer stemness [37, 38]. Our findings demonstrate that YAP is activated downstream of β-catenin signaling, underscoring the necessity of considering cellular context and various stimuli when dissecting the functional implications of these signaling pathways.

Cellular senescence is recognized as a hallmark of cancer, playing a complex role in tumor biology and progression [39]. While the senescence program was shown to stimulate immunosurveillance mechanisms that promote the elimination of tumor cells, it also contributes to chronic inflammation, drug resistance, and the potential emergence of cancer stem cell populations [40–42]. In our study, we elucidate a novel function of senescence in driving metastatic heterogeneity, thereby enhancing the aggressiveness of ovarian cancer. While traditionally viewed as a barrier to tumorigenesis due to its prevalence in pre-malignant lesions [43], emerging evidence highlights the multifaceted impact of senescence on cell fate, encompassing biological processes such as cell cycle regulation, chromatin remodeling, mitochondrial function, and cell-extrinsic effects [44]. Remarkably, senescence can be induced by various anticancer therapies, especially cytotoxic chemotherapies that cause DNA damage [45]. These therapy-induced senescent cells may play a critical role in shaping long-term treatment responses and influencing the tumor microenvironment.

Senescence is a pivotal phenomenon not only in anticancer therapy responses, but also tumor progression. Notably, NM cells demonstrate senescence-like characteristics even in the absence of therapeutic intervention. It is critical to differentiate between therapy-induced senescence and spontaneous senescence, as the clinical relevance of the latter in untreated cancer patients has been largely underestimated. Recent studies have begun to shed light on the presence of senescent tumor cells in untreated malignancies, including breast, thyroid, prostate, colorectal, and gastric cancers [46]. This underappreciation largely originates from technical challenges in identifying senescent cells within tumor samples and the tendency to regard spontaneously senescent tumor cells as artifacts arising from in vitro culture conditions [47]. Notably, recent investigations have identified naturally occurring senescent tumor cells in chemo-naive ovarian cancer patients [48]. Significantly, these spontaneously senescent cancer cells have been demonstrated to promote tumor progression more efficiently than those senescent cells induced by chemotherapy [49]. This evidence challenges the long-standing view that spontaneously arisen senescent tumor cells function solely as a tumor suppressor mechanism. Given their growth-arrested state, senescent tumor cells may inherently possess resistance to chemotherapeutic agents that predominantly target actively proliferating cells. Consequently, these cells may act as a reservoir of resistance, potentially increasing the risk of tumor recurrence post-therapy. Thus, elucidating the intrinsic drivers of spontaneous tumor senescence is critical for enhancing treatment efficacy and tailoring therapeutic strategies.

A pivotal aspect of senescence is the SASP, which is a key driver of age-related inflammation through the secretion of a milieu of SASP factors. These include cytokines, growth factors, and angiogenic factors, contributing to various age-related disabilities and increasing vulnerability to chronic diseases [50]. While SASP are well-recognized in the context of cancer, most research has focused on the SASP of the host stromal cells [51–53], rather than cancer cells themselves, as a source of these pro-inflammatory and pro-metastatic factors. Thus, the functional role of the SASP program in senescent cancer cells remains relatively underexplored, despite emerging evidence of its clinical relevance [54]. Here, we show for the first time that the SASP of senescent-like NM cells can cooperatively enhance metastasis of another subclone, providing important pathophysiological implications of the cancer cell SASP in the context of metastatic progression. Although contributions from non-SASP factors cannot be excluded, senescence-associated signaling appears to constitute one important element within a broader, multifactorial secretome rather than a single explanatory mechanism. Intriguingly, research has indicated significant heterogeneity in the senescence state among tumor cells, as evidenced by the diverse composition of SASP factors that could mediate divergent functional effects [55]. Whether the SASP profile of NM may evolve over time in response to environmental cues and how these alterations may further influence metastasis remains to be elucidated. Such insights could be critical for developing targeted therapeutic interventions based on SASP modulation.

Our findings demonstrate how β-catenin/YAP heterogeneity facilitates cooperation among tumor subclones through SASP, revealing actionable therapeutic vulnerabilities in ovarian cancer. Resistance to traditional cytotoxic therapies presents a significant challenge in oncology, which could be attributed to intrinsic apoptotic defects found in many tumors. However, tumors that retain intact senescence pathways may exhibit susceptibility to senolytic agents, offering a potential avenue for therapeutic intervention. We therefore propose a multi-pronged approach that leverages senotherapeutics to disrupt the pro-metastatic functions of SASP, thereby enhancing treatment efficacy and overcome existing resistance mechanisms. Fisetin, a natural flavonoid prevalent in various fruits and vegetables, has emerged as a promising agent due to its potent senolytic properties demonstrated in preclinical cancer models. Notably, fisetin has exhibited effectiveness both as a monotherapy and in combination with other anticancer agents [56]. Recent findings from randomized controlled clinical trials have indicated that fisetin can ameliorate inflammatory status in colorectal cancer patients undergoing chemotherapy, suggesting its potential as a complementary therapy [57]. Additionally, in vitro studies have shown that fisetin induces cell death in ovarian cancer cells, and advancements in nanodelivery systems may enhance its therapeutic efficacy in vivo [58]. Our study showed that fisetin could inhibit metastasis in mouse models, warranting further exploration of its clinical applicability in treating ovarian and other advanced cancers. In addition, reprogramming cancer cells to enter a senescence program has been proposed as a means to amplify the tumor-suppressive effects of senotherapies. Our data suggest that inhibiting the β-catenin/YAP pathway in HM cells could potentially facilitate this reprogramming.

Overall, our research underscores the necessity of addressing heterogeneity-driven progression in cancer pathology. This perspective shifts the focus from merely characterizing clonal heterogeneity to deciphering the functional networks that drive aggressive cancer phenotypes. Understanding these networks is essential for identifying new therapeutic strategies that can effectively target the unique characteristics of tumor subclones, ultimately improving patient outcomes in metastatic cancer.

## Methods

### Cell lines and cell culture

The HEYA8 NM and HM sublines were derived and cultured in RPMI medium (Invitrogen) as previously described [14]. Media were supplemented with 100 units/mL penicillin, 100 mg/mL streptomycin and 5% fetal bovine serum. All cultures were maintained in a humidified incubator at 37 °C with 5% CO_2_. NT and β-catenin KO HM cells were established using Edit-R predesigned all-in-one lentiviral sgRNA (Dharmacon) and the MISSION lentiviral packaging mix (Merck). Briefly, conditioned media containing the recombinant viruses were collected and filtered through 0.45 μm-pore-size filters. The filtrate was then used for transduction in the presence of 4 μg/ml polybrene (Sigma). Cells were selected with 1 μg/ml puromycin.

### Migration assay

Cells were seeded in 24-well transwell inserts containing serum-free medium and allowed to migrate toward complete medium or conditioned media for 16 hours. The migrated cells on the lower surface were fixed with ice-cold methanol and subsequently stained with 0.5% crystal violet. For assays requiring cell differentiation, cells were stained with CellTracker™ Green CMFDA or CellTracker™ Red CMTPX dye (Invitrogen) according to the manufacturer’s instructions before the assay. Migrated cells were then observed using fluorescence microscopy. Random fields from each well were captured at ×10 magnification, and migrated cells were quantified by ImageJ.

### Western blot analysis

Cells were lysed using cell lysis buffer (Cell Signaling Technology). Total proteins were separated by electrophoresis on 7.5% or 10% polyacrylamide gels and then transferred to nitrocellulose membranes. The membranes were blocked with non-fat dry milk for one hour and subsequently incubated with primary antibodies at 4 °C for 1 hour to overnight. After washing the membranes three times, they were incubated with the appropriate peroxidase-conjugated secondary antibodies (Bio-rad). Protein bands were detected using Western Lightning Plus Enhanced Chemiluminescence (Perkin Elmer, Waltham, MA), and their intensities were quantified by densitometry using ImageJ software. The following primary antibodies were used: β-catenin (#8480), total YAP (#14074), phospho-YAP (S127) (#13008), phospho-YAP (S397) (#13619), p21 (#2947), IL-6 (#12153), and PAI-1 (#11907) (all from Cell Signaling Technology), and β-actin (Sigma, #A5441). Blots were imaged using the ChemiDoc MP System (Bio-Rad).

### SA β-gal staining

SA β-gal expression was visualized using the senescence β-galactosidase staining kit (Cell Signaling Technology) following the manufacturer’s protocol. Briefly, cells were fixed for 15 min at room temperature. After washing with PBS, β-gal staining solution containing X-gal was added, and the plate was incubated at 37 °C overnight in a dry incubator for the development of blue color.

### Cytokine array

Conditioned media were analyzed using the Human Cytokine Array Kit (Panel A; R&D Systems) following the manufacturer’s instructions. This array measures the relative levels of 36 different cytokines and chemokines. In brief, cytokine array membranes were blocked with a blocking buffer for 1 hour and then incubated overnight at 4 °C with a sample/antibody mixture on a rocking platform shaker. The membranes were washed three times with wash buffer, followed by a 30-minute incubation with diluted streptavidin-HRP. After three additional washes, the proteins on the membranes were detected using chemiluminescence.

### In vivo studies

Animal studies were performed in accordance with protocols approved by the Committee on the Use of Live Animals in Teaching and Research at The University of Hong Kong, as well as the Animals (Control of Experiments) Ordinance of Hong Kong. Female nude or NSG mice were obtained from and kept at the Centre for Comparative Medicine Research (The University of Hong Kong). A total of two million cancer cells were injected intraperitoneally into the mice. For drug treatment, mice were randomly assigned to groups and received intraperitoneal injections of either a vehicle control (10% Tween 80, 10% DMSO in PBS), verteporfin, or fisetin (MedChemExpress) every other day for a total of five doses, beginning on Day 5 after tumor injection. For treatment with cell line-derived CM, 1 week after HM tumor inoculation, mice received intraperitoneal injections of concentrated control medium, concentrated HM-derived CM, or concentrated NM-derived CM (200 µl every two days) for a total duration of two weeks. To monitor intraperitoneal metastasis, the mice were given 200 μl of 30 mg/ml D-luciferin (Perkin Elmer), and imaging was conducted starting 10 minutes after the injection using a Xenogen IVIS 100 cooled CCD camera (Xenogen). At the end of the experiments, tumor tissues were fixed in formalin and embedded in paraffin. All in vivo experiments were performed in a non-blinded manner. Tumor spheroids or clusters >40 μm were enriched from ascitic fluid. To preserve cell-surface proteins, no additional sorting was performed. Immunostaining of PAX8 (Abcam, #ab191870), a biomarker expressed in ~90% of high-grade serous ovarian cancer, confirmed that these spheroids were predominantly composed of tumor cells ( > 70%) (Fig. S4B). The spheroid fraction was then analyzed as a whole, providing gene and protein expression profiles that primarily reflect tumor-derived signals.

### Patient tumors

Formalin-fixed and paraffin-embedded clinical samples of paired ovarian high-grade serous carcinoma and its metastasis were obtained from the pathology archives of the Department of Pathology, The University of Hong Kong, Queen Mary Hospital, for immunohistochemical analyses.

### Immunohistochemistry

FFPE sections from patient or mice tumors were deparaffinized with xylene and rehydrated in graded ethanol. After blocking and heat-induced antigen retrieval using citrate buffer, the specimens were separately incubated with primary antibody overnight at 4 °C. The slides were further processed using rabbit-specific HRP/DAB (ABC) detection IHC kit according to the manufacturer’s protocol (Abcam), followed by hematoxylin counterstaining. Slides were scanned on an Akoya Vectra Polaris scanner at 320 magnification, and semi-quantitative analysis of β-catenin (#8480), p21 (#2947), YAP (#14074) (all from Cell Signaling Technology), Active YAP (Abcam, #ab205270), and Ki67 (Abcam, #ab16667) expression was performed using QuPath 0.4.4 software. Two representative fields per specimen with the strongest marker expression were selected for positive cell detection and tumor cell classification. After classification and positive cell detection, the percentage of tumor cells positive for each marker was quantified and analyzed in GraphPad Prism. Data are presented as percentage of marker-positive tumor cells.

### Statistical analysis

All experiments were repeated at least twice in duplicate. Each of them yields essentially similar results. The significance of the data was analyzed by unpaired Student’s *t* test for comparison between two groups or one-way ANOVA followed by a Tukey’s test for comparison between three or more groups. *P* < 0.05 was considered significant.

## Supplementary information

Supplemental Figures

Raw blots

## Data Availability

All data generated or analysed during this study are included in this published article and its supplementary information files.
