# Supplementary material for: Subclonal β-catenin/YAP signaling heterogeneity accelerates ovarian cancer metastasis through a senescence-associated secretory phenotype
Source: Cell Death Dis. 2026 Apr 23;17(1):539. doi: 10.1038/s41419-026-08737-7 (PMC13237159; doi:10.1038/s41419-026-08737-7)
Supplement: Supplementary file 1 — Supplemental Figures [file 41419_2026_8737_MOESM1_ESM.pptx]

## Slide 1
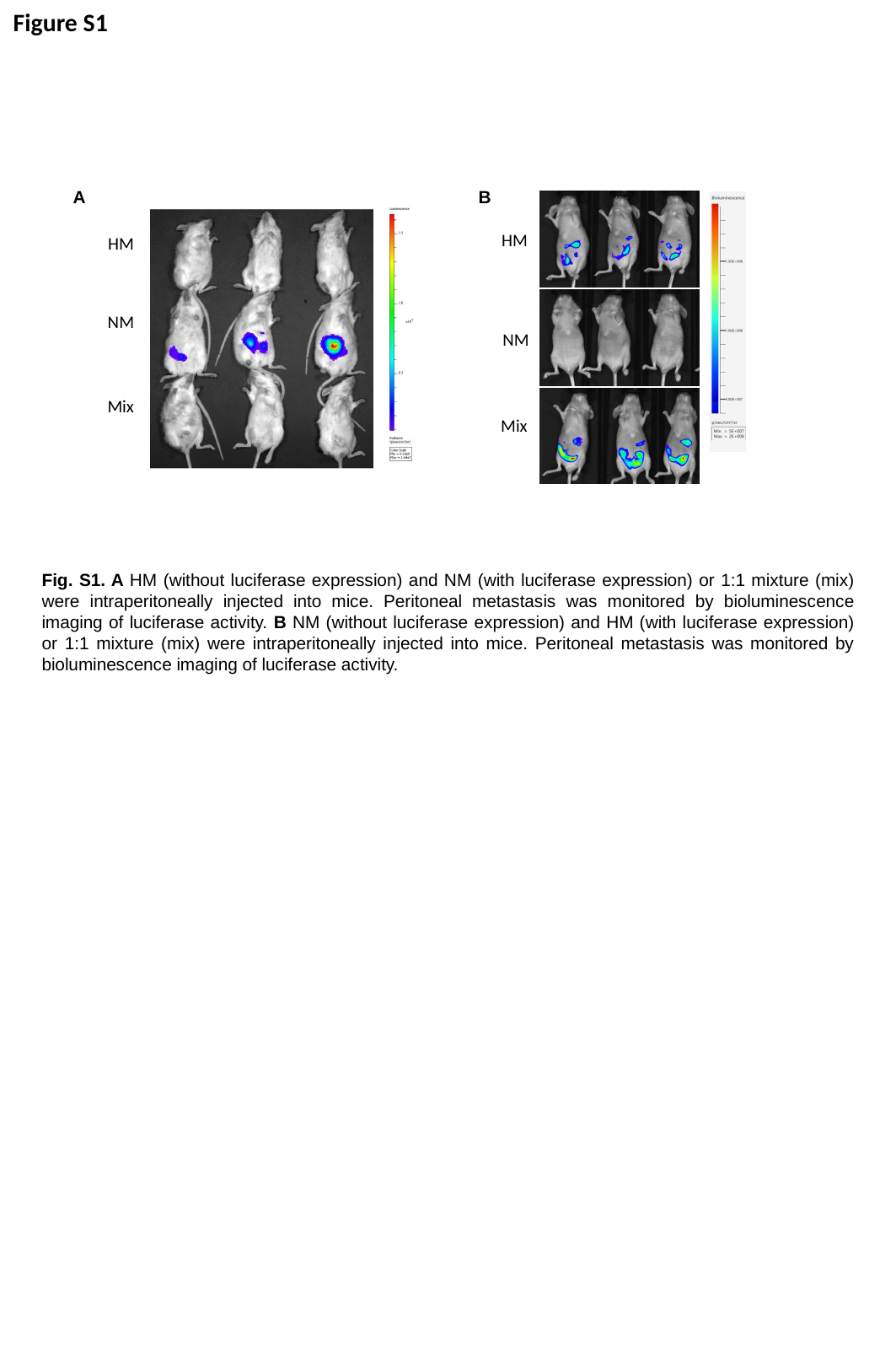

Figure S1
A
B
HM
HM
NM
NM
Mix
Mix
Fig. S1. A HM (without luciferase expression) and NM (with luciferase expression) or 1:1 mixture (mix) were intraperitoneally injected into mice. Peritoneal metastasis was monitored by bioluminescence imaging of luciferase activity. B NM (without luciferase expression) and HM (with luciferase expression) or 1:1 mixture (mix) were intraperitoneally injected into mice. Peritoneal metastasis was monitored by bioluminescence imaging of luciferase activity.

## Slide 2
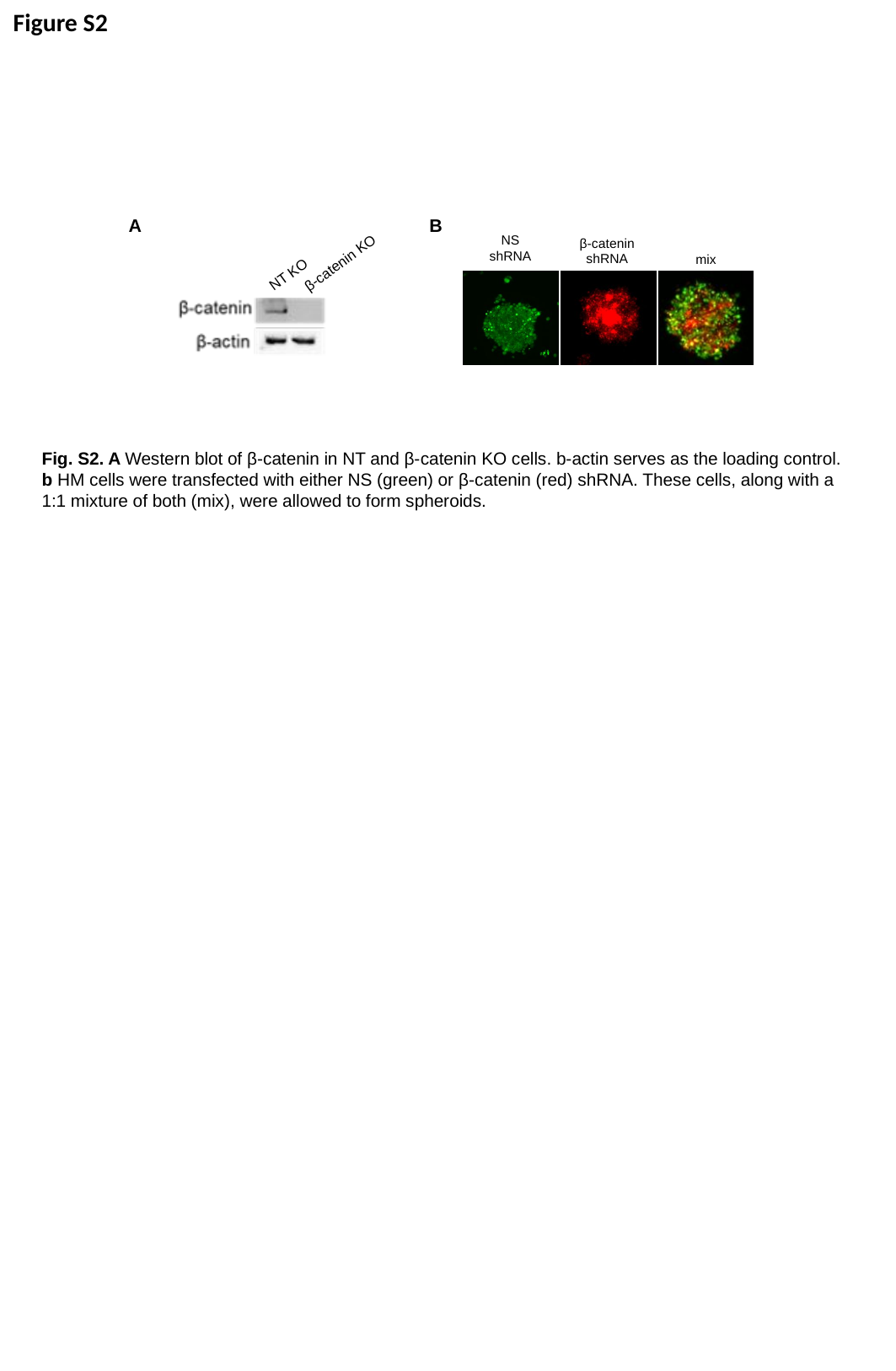

Figure S2
A
B
NS
shRNA
β-catenin shRNA
mix
β-catenin KO
NT KO
Fig. S2. A Western blot of β-catenin in NT and β-catenin KO cells. b-actin serves as the loading control. b HM cells were transfected with either NS (green) or β-catenin (red) shRNA. These cells, along with a 1:1 mixture of both (mix), were allowed to form spheroids.

## Slide 3
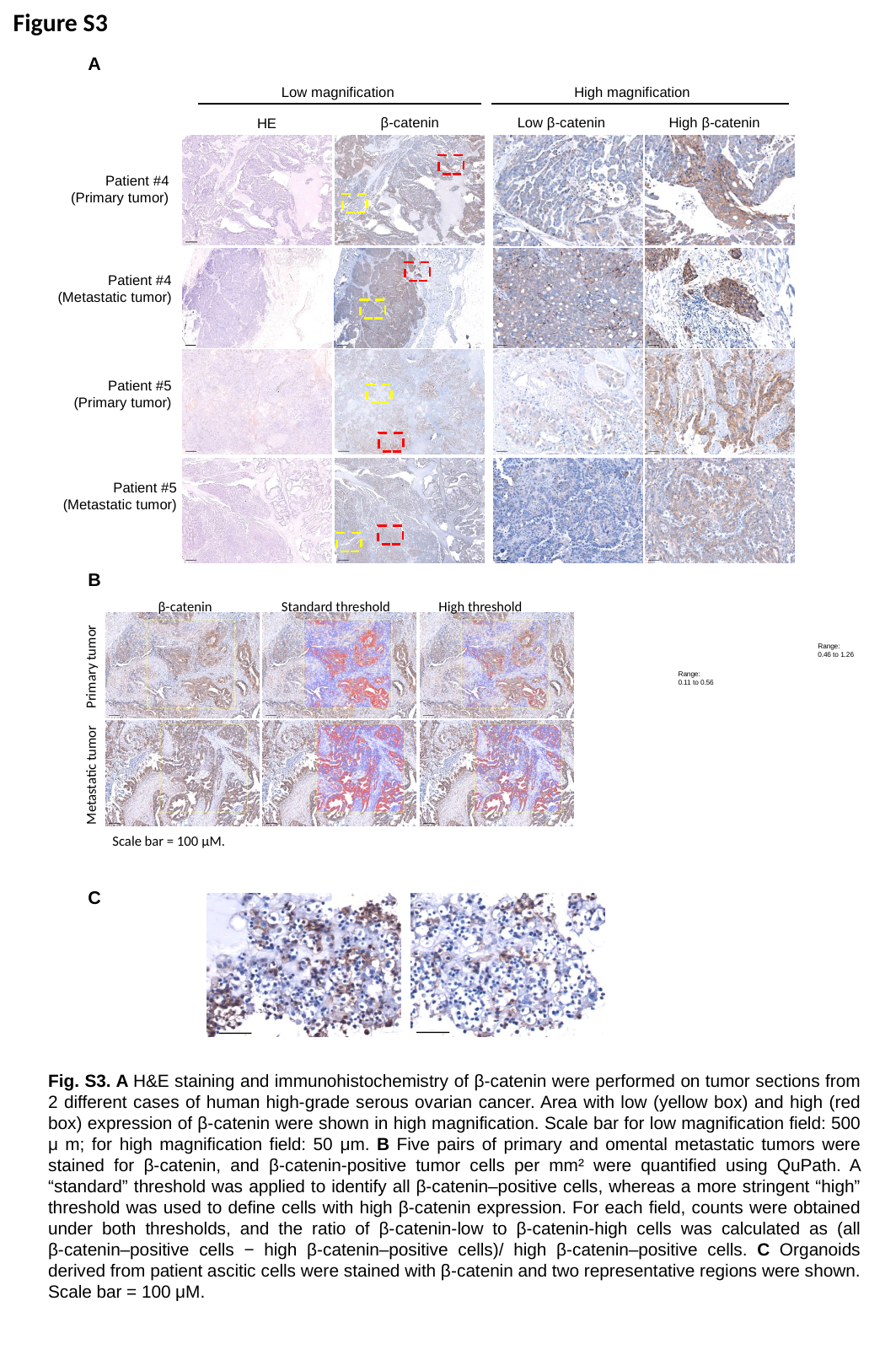

Figure S3
A
Low magnification
High magnification
β-catenin
Low β-catenin
High β-catenin
HE
Patient #4
(Primary tumor)
Patient #4
(Metastatic tumor)
Patient #5
(Primary tumor)
Patient #5
(Metastatic tumor)
B
Standard threshold
High threshold
β-catenin
Primary tumor
Metastatic tumor
Scale bar = 100 μM.
Range:
0.46 to 1.26
Range:
0.11 to 0.56
C
Fig. S3. A H&E staining and immunohistochemistry of β-catenin were performed on tumor sections from 2 different cases of human high-grade serous ovarian cancer. Area with low (yellow box) and high (red box) expression of β-catenin were shown in high magnification. Scale bar for low magnification field: 500 μ m; for high magnification field: 50 μm. B Five pairs of primary and omental metastatic tumors were stained for β‑catenin, and β‑catenin‑positive tumor cells per mm² were quantified using QuPath. A “standard” threshold was applied to identify all β‑catenin–positive cells, whereas a more stringent “high” threshold was used to define cells with high β‑catenin expression. For each field, counts were obtained under both thresholds, and the ratio of β‑catenin‑low to β‑catenin‑high cells was calculated as (all β‑catenin–positive cells − high β‑catenin–positive cells)/ high β‑catenin–positive cells. C Organoids derived from patient ascitic cells were stained with β-catenin and two representative regions were shown. Scale bar = 100 μM.

## Slide 4
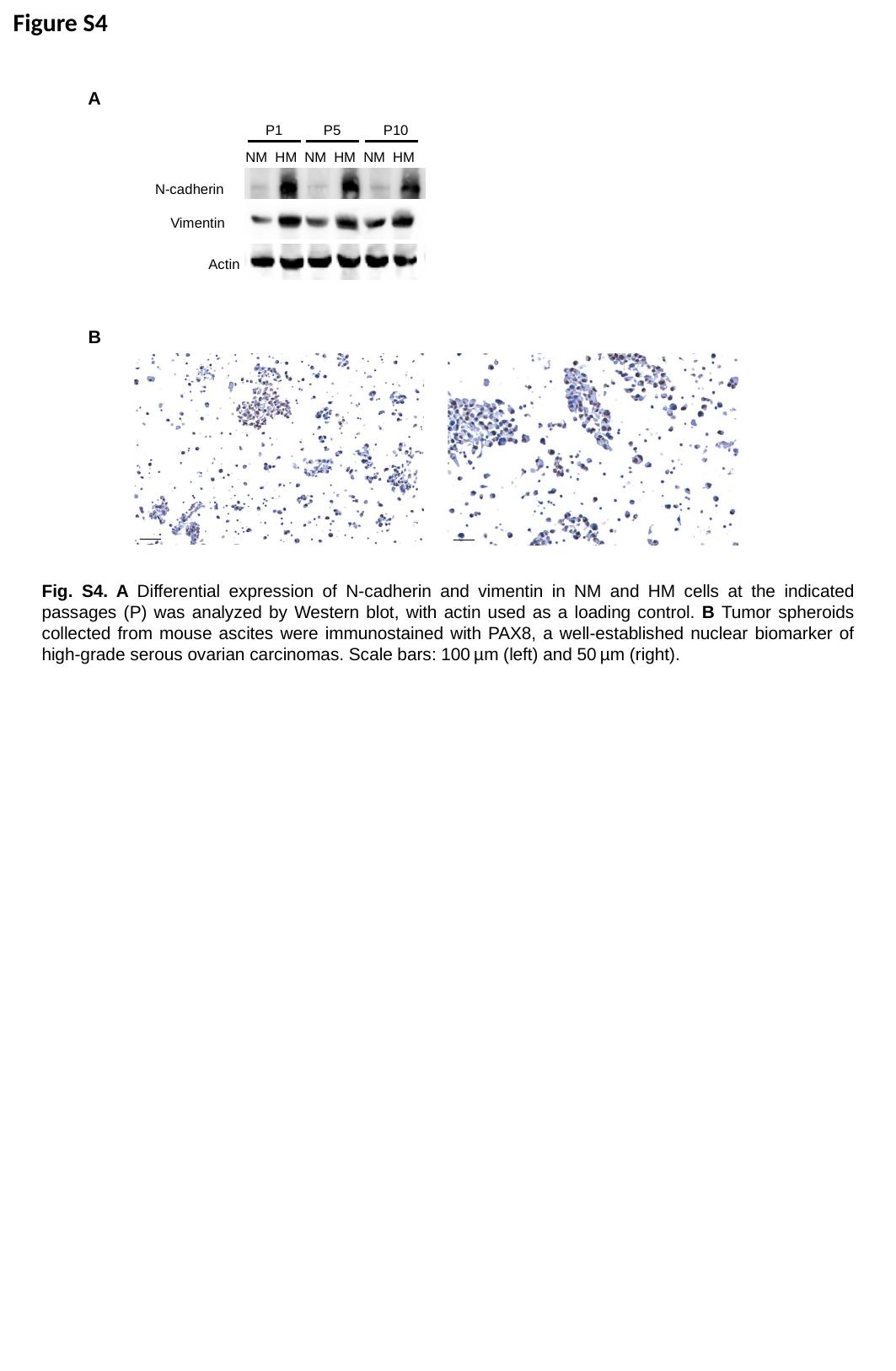

Figure S4
A
P1
P5
P10
NM
HM
NM
HM
NM
HM
N-cadherin
Vimentin
Actin
B
Fig. S4. A Differential expression of N-cadherin and vimentin in NM and HM cells at the indicated passages (P) was analyzed by Western blot, with actin used as a loading control. B Tumor spheroids collected from mouse ascites were immunostained with PAX8, a well-established nuclear biomarker of high-grade serous ovarian carcinomas. Scale bars: 100 µm (left) and 50 µm (right).

## Slide 5
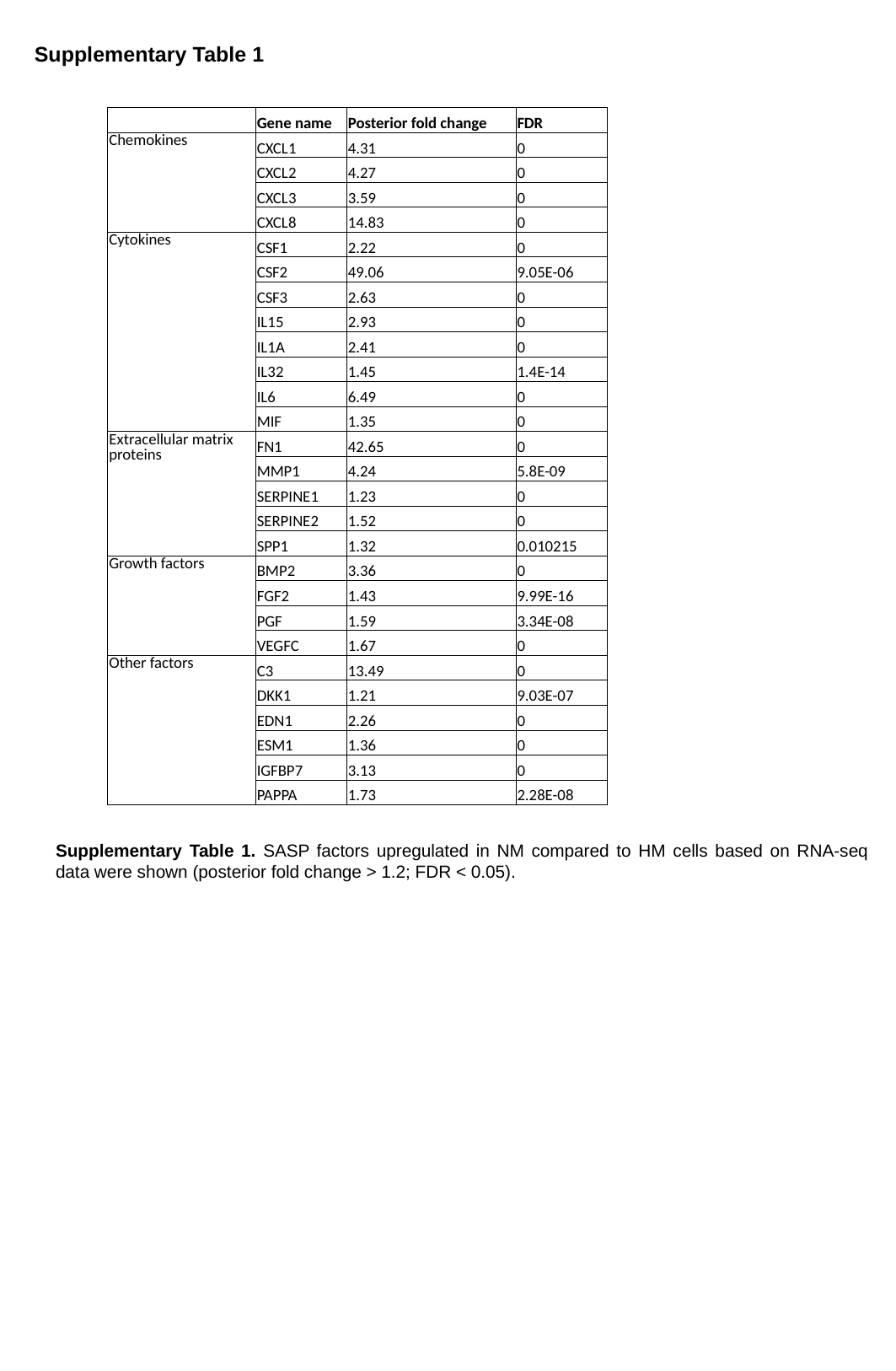

Supplementary Table 1
| | Gene name | Posterior fold change | FDR |
| --- | --- | --- | --- |
| Chemokines | CXCL1 | 4.31 | 0 |
| | CXCL2 | 4.27 | 0 |
| | CXCL3 | 3.59 | 0 |
| | CXCL8 | 14.83 | 0 |
| Cytokines | CSF1 | 2.22 | 0 |
| | CSF2 | 49.06 | 9.05E-06 |
| | CSF3 | 2.63 | 0 |
| | IL15 | 2.93 | 0 |
| | IL1A | 2.41 | 0 |
| | IL32 | 1.45 | 1.4E-14 |
| | IL6 | 6.49 | 0 |
| | MIF | 1.35 | 0 |
| Extracellular matrix proteins | FN1 | 42.65 | 0 |
| | MMP1 | 4.24 | 5.8E-09 |
| | SERPINE1 | 1.23 | 0 |
| | SERPINE2 | 1.52 | 0 |
| | SPP1 | 1.32 | 0.010215 |
| Growth factors | BMP2 | 3.36 | 0 |
| | FGF2 | 1.43 | 9.99E-16 |
| | PGF | 1.59 | 3.34E-08 |
| | VEGFC | 1.67 | 0 |
| Other factors | C3 | 13.49 | 0 |
| | DKK1 | 1.21 | 9.03E-07 |
| | EDN1 | 2.26 | 0 |
| | ESM1 | 1.36 | 0 |
| | IGFBP7 | 3.13 | 0 |
| | PAPPA | 1.73 | 2.28E-08 |
Supplementary Table 1. SASP factors upregulated in NM compared to HM cells based on RNA-seq data were shown (posterior fold change > 1.2; FDR < 0.05).
