# Supplementary material for: Subclonal β-catenin/YAP signaling heterogeneity accelerates ovarian cancer metastasis through a senescence-associated secretory phenotype
Source: Cell Death Dis. 2026 Apr 23;17(1):539. doi: 10.1038/s41419-026-08737-7 (PMC13237159; doi:10.1038/s41419-026-08737-7)
Supplement: Supplementary file 2 — Raw blots [file 41419_2026_8737_MOESM2_ESM.pptx]

## Slide 1
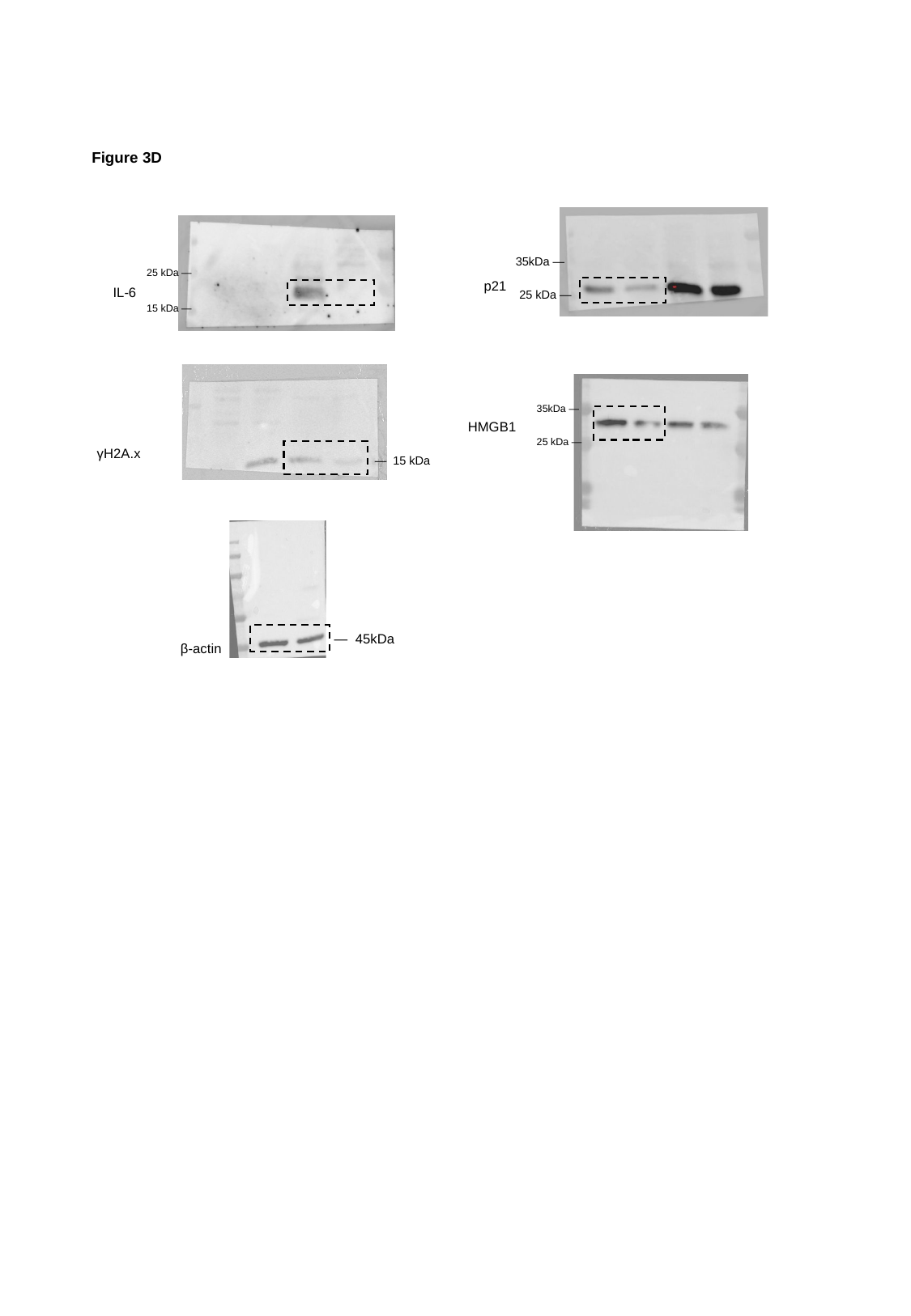

Figure 3D
p21
35kDa —
25 kDa —
25 kDa —
IL-6
15 kDa —
γH2A.x
— 15 kDa
HMGB1
35kDa —
25 kDa —
β-actin
— 45kDa

## Slide 2
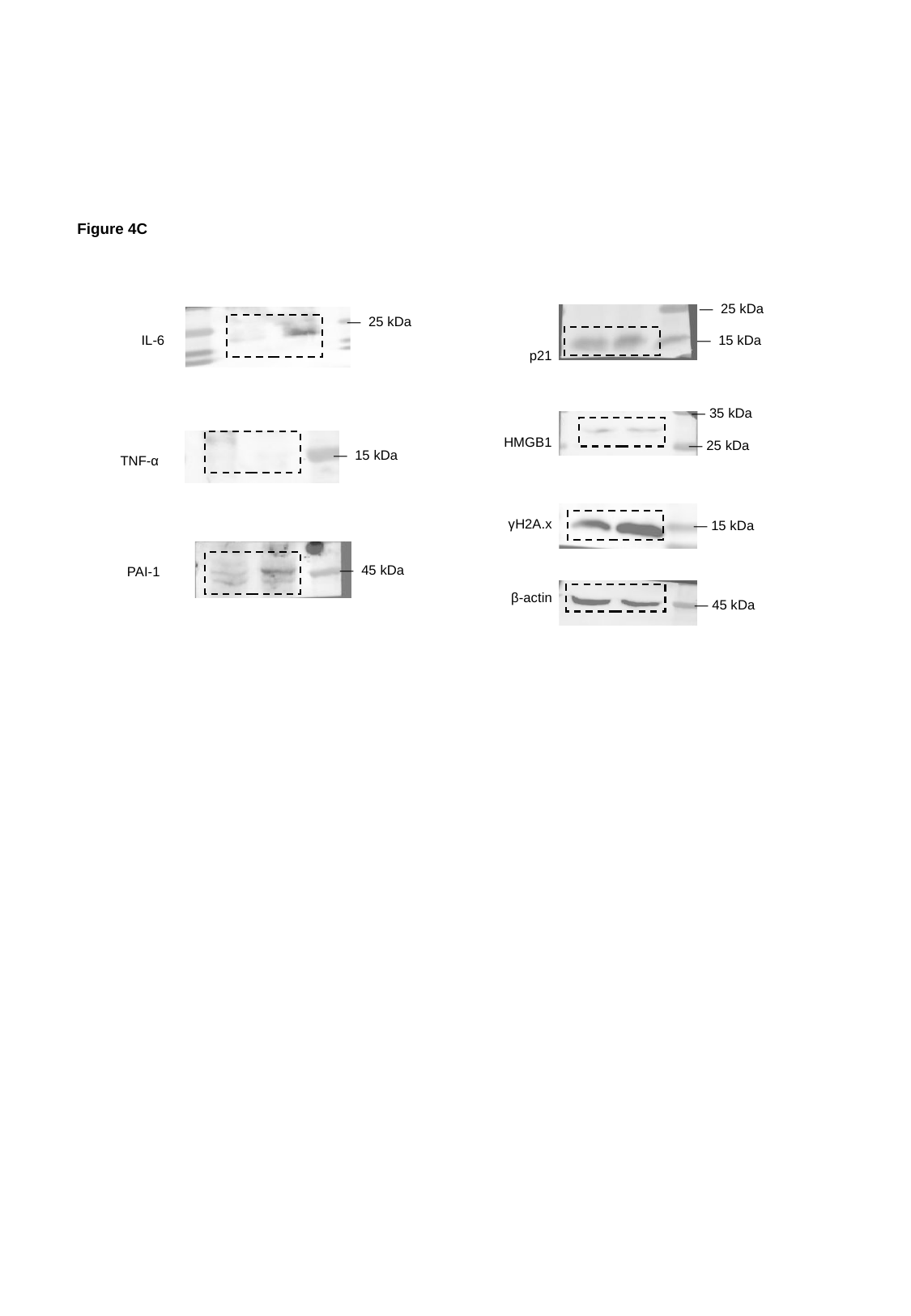

Figure 4C
— 25 kDa
— 25 kDa
IL-6
— 15 kDa
p21
— 35 kDa
HMGB1
— 25 kDa
— 15 kDa
TNF-α
γH2A.x
— 15 kDa
— 45 kDa
PAI-1
β-actin
— 45 kDa

## Slide 3
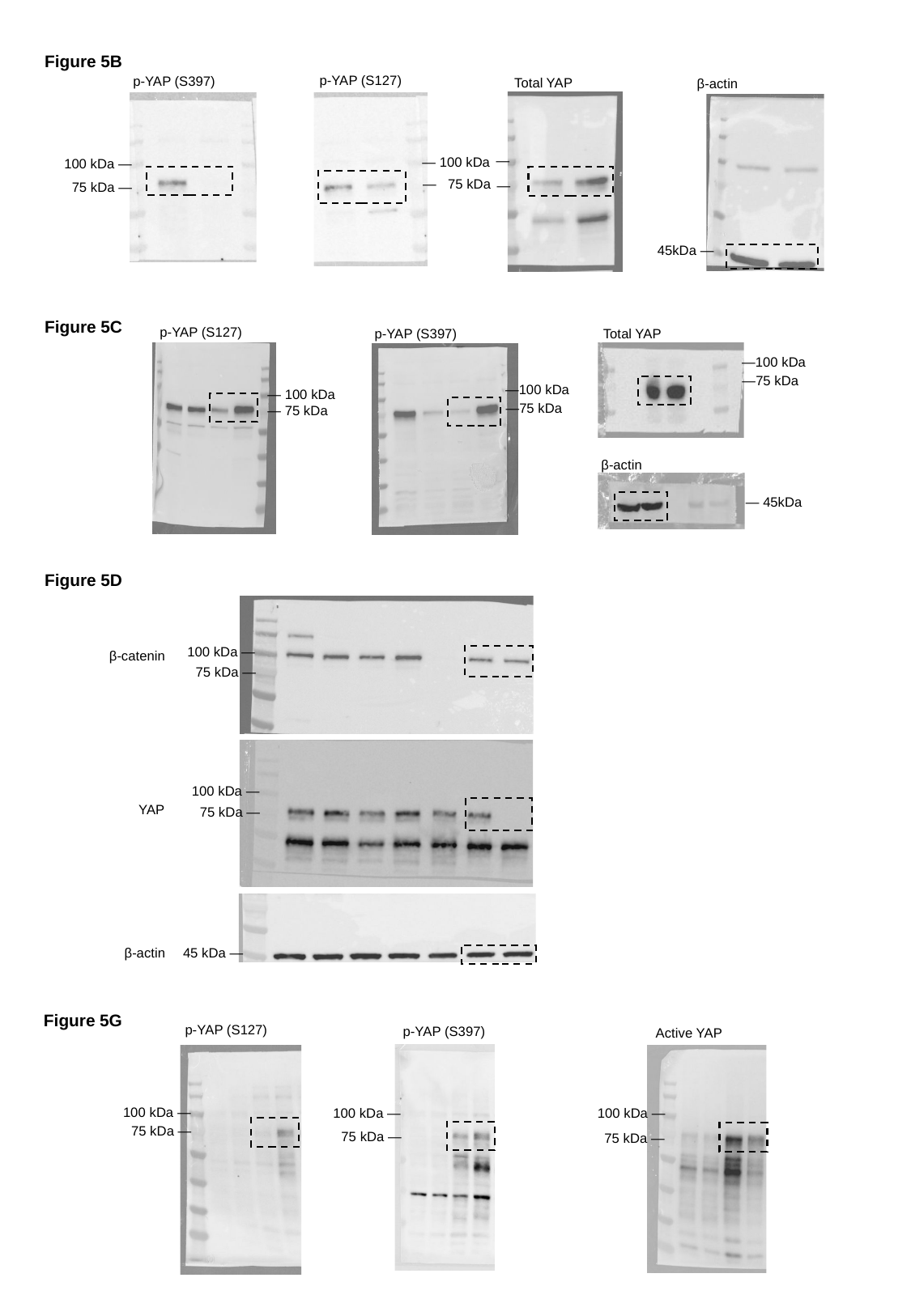

Figure 5B
p-YAP (S127)
p-YAP (S397)
Total YAP
β-actin
—
— 100 kDa
100 kDa —
— 75 kDa
—
75 kDa —
45kDa —
Figure 5C
p-YAP (S127)
— 100 kDa
— 75 kDa
Total YAP
p-YAP (S397)
—100 kDa
—75 kDa
—100 kDa
—75 kDa
β-actin
— 45kDa
Figure 5D
100 kDa —
β-catenin
75 kDa —
100 kDa —
YAP
75 kDa —
45 kDa —
β-actin
Figure 5G
p-YAP (S127)
p-YAP (S397)
100 kDa —
75 kDa —
Active YAP
100 kDa —
100 kDa —
75 kDa —
75 kDa —
